# Vitamin B12: A Comprehensive Review of Natural vs Synthetic Forms of Consumption and Supplementation

**DOI:** 10.7759/cureus.96258

**Published:** 2025-11-06

**Authors:** Christopher R Behringer, Amulya Kulkarni, Alisha Weinstein

**Affiliations:** 1 Clinical Education, Lake Erie College of Osteopathic Medicine, Erie, USA; 2 Lifestyle Medicine/Family Medicine, Owensboro Health, Owensboro, USA; 3 Neurology, Lake Erie College of Osteopathic Medicine, Erie, USA; 4 Family Medicine, Lake Erie College of Osteopathic Medicine, Erie, USA

**Keywords:** natural vs synthetic, supplement, vitamin b 12, vitamin deficiency, vit b12 deficiency

## Abstract

Vitamin B12, also known as cyanocobalamin, methylcobalamin, and adenosylcobalamin, is an essential vitamin that can be primarily obtained through the human diet. Although animal-based foods supply adequate B12 for most people, deficiency remains common in older adults, vegetarians and vegans, and in those with gastrointestinal disease or malabsorption. Deficiency can lead to megaloblastic anemia, neurologic symptoms, and pregnancy complications. In healthy individuals, supplementation raises serum B12 levels to a similar extent as dietary intake; however, in disease states such as megaloblastic anemia, supplementation is required because diet alone is not sufficient. Interest has grown in how natural dietary forms, such as methylcobalamin, compare with the widely used synthetic form, cyanocobalamin, with respect to absorption, metabolism, and clinical effect. This comprehensive review summarizes B12 physiology, sources, absorption, and the clinical picture of deficiency, and compares natural and synthetic forms in common patient scenarios. We also note practical questions for future work, including long-term outcomes of supplementation and whether prophylaxis is useful in high-risk groups.

## Introduction and background

Introduction

Vitamin B12 has a wide range of benefits in the human body. It is used in red blood cell formation, cellular metabolism, cellular energy production, DNA synthesis, and myelination. B12 serves as a cofactor for enzymes that assist in the creation of methylmalonyl-coenzyme A (CoA)** **mutase and methionine, an enzyme required for metabolic processes and tetrahydrofolate production. Several reactions require B12 to properly produce purines and pyrimidines, which act as the building blocks of DNA. This DNA is then used to produce reticulocytes, which eventually develop into red blood cells. Disrupting these reactions due to vitamin B12 deficiency leads to one of many conditions known as megaloblastic anemia [1]. Clinically, deficiency manifests as macrocytic anemia, neuropathy, and cognitive changes, most often arising from malabsorption or impaired utilization rather than inadequate intake [1].

Additionally, reviews by the National Institutes of Health showed that B12 supplementation may play a role in neurologic conditions. Observational and interventional evidence suggest that vitamin B12 status influences depressive symptoms, and adjunctive supplementation can enhance antidepressant response in certain patients [2]. For example, there is evidence that B12 may delay the onset of depression as well as improve the effects of antidepressant medication [2]. Mechanistic studies link disrupted one-carbon metabolism, elevated homocysteine, and mitochondrial dysfunction in B12 deficiency to pathways implicated in Alzheimer’s disease, supporting investigation of B12 in neurodegeneration [3]. Moreover, B12 deficiency can lead to demyelination of the lateral and posterior columns, resulting in subacute combined degeneration. It is involved in regulating the immune system and may confer a benefit for antiviral immunity [4]. Recent reviews highlight vitamin B12’s broader contribution to immune homeostasis and antiviral defense, underscoring its systemic importance beyond hematologic and neurologic functions [4]. Vitamin B12 is also thought to be essential in the function of the skeletal muscle-gut-brain axis, contributing to muscle strength, neurobehavioral conditions, and the gut microbiome [4]. The function of B12 and potential disease outcomes due to deficiency are summarized in Table 1.

**Table 1:** Vitamin B12 function with the potential disease outcome due to deficiency This table is original and created by the authors.

Vitamin B12 Function	Potential Disease if Vitamin B12 Deficient
Myelination	Subacute combined degeneration
DNA synthesis	Megaloblastic anemia
Red blood cell production	Megaloblastic anemia
Cofactor for methionine	Lack of purine and pyrimidines
Antiviral immunity	Reduced immunity response
Skeletal muscle-gut-brain axis	Muscle weakness, gastrointestinal issues, and neuropsychiatric disorders

## Review

Sources and recommended amount of B12

There are a variety of sources of vitamin B12, both supplemental and natural. The highest microgram per serving of natural B12 is found in foods such as beef liver, clams, fortified nutritional yeast, salmon, Greek yogurt, and eggs [5-7]. This is summarized in Table 2.

**Table 2:** Vitamin B12 content of foods in micrograms per serving This table is original and created by the authors. Based on information available in O'Leary and Samman [1] and Lauer et al. [3].

Food with B12	Micrograms of B12 per Serving
Beef liver	70.7 mcg
Clams	17 mcg
Fortified nutritional yeast	8.3–24 mcg
Salmon	2.6 mcg
Nonfat plain Greek yogurt	1.3 mcg
Egg	0.5 mcg

The National Institute of Health has published the daily recommended B12 intake for different populations. For adult males and females, they recommended 2.4 mcg/day, for pregnant females 2.6 mcg/day, and lactating females 2.8 mcg/day [5]. The NIH also notes that infants need the least amount of B12, and this requirement increases as the child ages [5].

B12 deficiency and susceptible populations

The general population ingests sufficient B12 in their diet; however, certain groups of individuals may be more susceptible [7]. According to the Mayo Clinic, vegetarians, vegans, individuals with digestive tract conditions that affect the absorption of B12, and the elderly are at an increased risk of B12 deficiency [8]. Additionally, it can be caused by either a lack of B12 in the diet, gastritis, pernicious anemia, digestive diseases (e.g., Crohn's disease and celiac disease), surgery on the GI tract, alcohol use disorder, transcobalamin II deficiency, or Sjögren's syndrome [9]. There are also various medications like metformin, proton pump inhibitors, histamine H2 blockers, and oral birth control that have the potential to affect B12 levels [9].

While most of the population ingests sufficient B12 levels, the following statistics estimate the percentage of individuals who have a B12 deficiency in specific age groups: 3% of people 20-39 years old, 4% of people 40-59 years old, and 6% of people 60+ years old [9].

Symptoms and diagnosis of a B12 deficiency

Presentation of a B12 deficiency can include anemia, fatigue, muscle weakness, nerve damage, mood disturbances, intestinal problems, and dementia [8]. Additionally, the following symptoms can develop slowly and may not be debilitating at first: sore mouth or tongue, yellow skin, weight loss, numbness, and tingling in hands and feet, and vision issues [9]. B12 is also involved in proper brain functioning, with deficiency leading to depression and paranoia as possible symptoms [9]. In pregnant individuals, it can lead to neural tube defects such as spina bifida, anencephaly, and encephalocele [1].

While these are the common symptoms, presentation will vary between patients, and if a B12 deficiency is suspected, a B12 blood level test should be obtained. If the B12 blood level is less than 150/mL, it is clinically considered a B12 deficiency [9].

A B12 deficiency can be treated with supplementation. However, if B12 deficiency is left untreated, serious and possibly life-threatening consequences may ensue. Peripheral neuropathy, degeneration of the spinal cord, paralysis, bowel incontinence, erectile dysfunction, depression, and paranoia may occur [9]. If the B12 deficiency is caused specifically by pernicious anemia, the patient may develop stomach cancer, birth defects such as spina bifida, anencephaly, or encephalocele [10].

Absorption of B12

The molecular configuration in which Vitamin B12 is presented to the body is a pertinent indicator of how efficiently it is absorbed into our GI tract [11]. Vitamin B12, also termed cobalamin, is a polar molecule that must bind specific transport proteins to enter enterocytes. Vitamin B12 in food is bound to proteins and must be detached before it is absorbed in the distal ileum [5].

Metabolism of B12 begins in the mouth as saliva breaks down the food [5]. Before ileal absorption, more protein-bound Vitamin B12 must undergo proteolytic cleavage through the functional activity of pepsin [11]. Chief cells within the epithelium of the stomach secrete pepsinogen, a digestive pro-enzyme. Hydrochloric acid in the stomach aids in the conversion of pepsinogen to pepsin, its active enzyme form [11]. Pepsin can then degrade the ingested protein complex to Vitamin B12, releasing the B12 to bind to haptocorrin. Haptocorrin (R-binder) is a glycoprotein released in saliva, bile, and pancreatic fluid and plays an essential role in the protection of B12 from the acidic environment of the stomach [12]. The haptocorrin-B12 complex is known as holohaptocorrin and is the predominant form delivered to the duodenum (12). Haptocorrin is then degraded by pancreatic enzymes termed proteases in the second part of the duodenum, allowing B12 to complex with intrinsic factor, a glycoprotein released by parietal cells in the stomach [11]. This intrinsic factor-B12 complex continues towards the distal segment of the ileum, where it is absorbed through receptor-mediated endocytosis into the enterocytes [5]. When vitamin B12 leaves the enterocytes and is introduced to the portal plasma, it binds to transcobalamin II, a non-glycoprotein secretory protein imperative in the transport of plasma vitamin B12 to bone marrow and multiple different tissues in the body [13].

**Figure 1:**
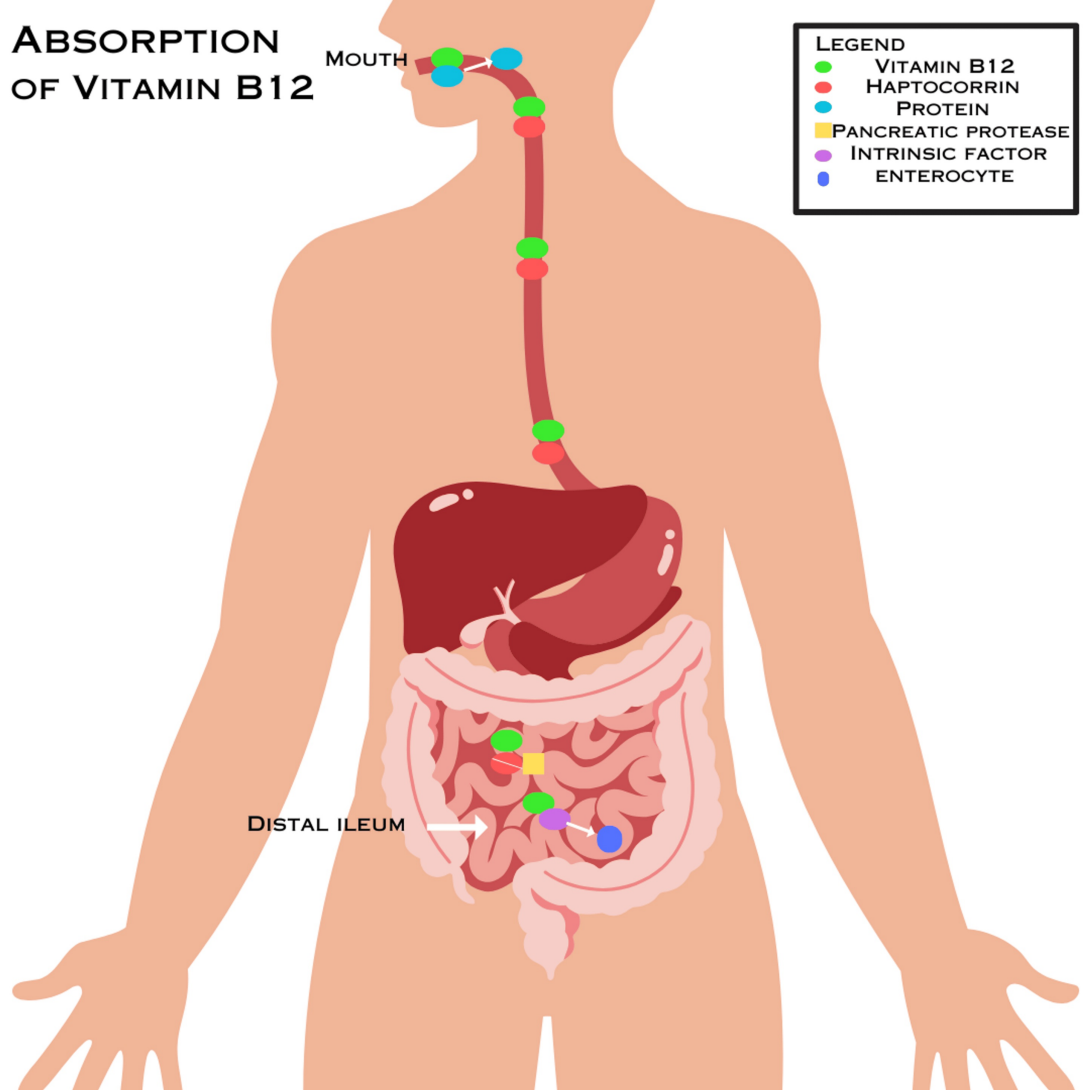
Absorption of B12 along the GI tract This figure was designed by Amulya Kulkarni. Images courtesy of Canva under Creative Common Licenses.

In summary, the absorption of Vitamin B12 starts in the mouth as it detaches from proteins in food by saliva and stomach acid and attaches to the R-binder protein haptocorrin, forming holohaptocorrin. As the holohaptocorrin complex makes its way to the duodenum, the haptocorrin is broken down by pancreatic protease, and the vitamin B12 is free to bind intrinsic factors and be absorbed into the enterocyte lining of the distal ileum. The absorption of B12 along the GI tract is summarized in Figure 1.

Methods

To gather relevant scientific literature, a comprehensive search was conducted using PubMed, Google Scholar, and government databases such as the NIH Office of Dietary Supplements. The search aimed to identify studies and reports related to vitamin B12, including its physiological roles, absorption mechanisms, clinical manifestations of deficiency, and the comparative effectiveness of supplementation methods. The time frame for inclusion spanned from 1999 to 2024. Priority was given to peer-reviewed studies such as randomized controlled trials, systematic reviews, and meta-analyses. Authoritative sources from recognized institutions (e.g., Mayo Clinic and Cleveland Clinic) were also included to support clinical perspectives. Search terms combined phrases such as “Vitamin B12”, “methylcobalamin”, “cyanocobalamin”, “B12 deficiency”, “neurological symptoms”, and “pernicious anemia”. Studies were first screened by reviewing titles and abstracts, and those determined as relevant were then read in full. Articles were excluded if they were opinion-based, anecdotal, non-clinical, or derived from promotional or non-academic sources. During review, attention was given to study design, population characteristics, intervention type, and measurable outcomes such as serum B12 levels or clinical symptom resolution. In total, 21 articles were included for this review.

B12 supplement (cyanocobalamin) vs natural B12 (methylcobalamin)

A study conducted in Los Angeles, California, presented evidence stating all forms of vitamin B12 in food and supplements are reduced to a common cobalamin molecule, which is then transformed into two active forms in cells, methylcobalamin and adenosyl cobalamin [14]. These forms are found to be bioidentical to the natural form of vitamin B12 and are preferred compared to a widely utilized synthetic form added to supplements, cyanocobalamin [14]. The synthetic form of vitamin B12, cyanocobalamin, has been utilized through oral supplements and injections [15]. The form by which this supplement is administered is largely dependent on the presentation of the patient. Although all three forms of vitamin B12 are shown to improve vitamin B12 status in patients with deficiencies, cyanocobalamin must first be broken down into cobalamin and cyanide to be further converted to active forms of B12 that are usable by the human body [15]. This mechanism of action may not occur in patients with SNPs (single-nucleotide polymorphisms) in the Vitamin B12 metabolic pathway [15].

Other animal studies showed that the urinary excretion of cyanocobalamin was three times higher compared to methylcobalamin, leading to the conclusion that methylcobalamin in its natural form led to 13% more cobalamins being stored in the liver than its synthetic form, cyanocobalamin [15]. Moreover, methylcobalamin is essential for cell growth and replication, and in many cases, the liver cannot convert large amounts of cyanocobalamin to the form that is needed for proper neuronal signaling and its full neuroprotective effects [15]. In addition, the methyl group in methylcobalamin has been studied for its role in serotonin production, a neurotransmitter that is key in the brain’s defense against excitotoxins [15]. Based on these findings, we recommend prioritizing methylcobalamin over cyanocobalamin, whether through diet or supplementation.

Megaloblastic anemia and B12 supplementation

Megaloblastic anemia is a type of macrocytic anemia that causes red blood cells to become abnormally large and to not properly divide, leading to a decreased amount. The size of the RBC does not allow it to leave the bone marrow and enter the blood, again decreasing the number of RBCs in the blood and ultimately causing anemia [16]. Several causes contribute to this diagnosis, such as pernicious anemia, B12 malabsorption, and a diet that lacks B12.

According to the current research, patients with megaloblastic anemia may consider methylcobalamin for supplementation over other sources [17]. This may be beneficial for two reasons: the first being that methylcobalamin will bypass several absorption steps, leading to more bioavailability. Secondly, methylcobalamin can be converted into S-adenosylmethionine, an important molecule in autophagy and several metabolic pathways [17] [18].

Before starting treatment, the clinician needs to evaluate the absorption levels of the patient through measurement of plasma B12 levels to determine if oral supplementation is appropriate [17]. If absorption levels are high, then oral is a plausible route and most cost-effective. If absorption levels are low, oral supplementation may not be effective, and the patient may opt for intramuscular injections [17]. 

Infection and B12

In addition to numerous functions, vitamin B12 also offers immune support for the body. B12 is integral to red blood cell (RBC) production and has also been proven to act as an immunomodulator for cellular immunity. During erythropoiesis, vitamin B12 and vitamin B9 are required for the proper differentiation and maturation of red blood cells. A deficiency in either vitamin inhibits purine and thymidylate synthesis, leading to defective DNA synthesis, erythroblast apoptosis, and anemia [19]. B12 serves as a cofactor for methionine synthesis by assisting in the conversion of homocysteine to methionine via methyl transfer [19]. The tetrahydrofolate from this reaction plays a crucial role in the production of purines required for DNA synthesis and the development of reticulocytes [19].

Recent studies have challenged the traditional belief that red blood cells are immunologically inert. During a study conducted by Minton, results suggested that in critically ill patients with conditions such as sepsis, RBCs showed increased toll-like receptor 9 expression and mitochondrial DMA (mtDNA) binding, leading to erythrophagocytosis and innate immune activation [20]. Furthermore, RBCs from healthy donors were shown to bind bacterial genomic DNA from bacterial and parasitic organisms, sequestering harmful cell-free DNA [20]. In addition to its role in erythropoiesis, B12 plays a direct role in increasing lymphocytes, such as CD8 T cells, and increasing the activity of natural killer (NK) cells in patients [21]. Decreased numbers of lymphocytes, CD8 T cells, suppressed NK cell activity, and abnormally high CD4/CD8 ratios were recorded in the control group with B12 deficiencies [21]. Methyl-B12 treatment balanced the high CD4/CD8 T cell count and increased NK cell activity, increasing immunity [21]. It has also been reported that vitamin B12 deficiency causes suppression of immune responses to bacteria and viruses in animal models [21]. By playing a significant role in RBC development and cellular immunity, B12 is a key regulator of our immune system and continues to be a sentinel player in protection against infection.

Vitamin B12 and severe acute respiratory syndrome coronavirus 2 (SARS-CoV-2)

Recent data has shown that vitamin B12 can help fight viral infections by altering the immune response. COVID-19 is a virus that presents with a variety of acute symptoms, the most common being shortness of breath, chest pain, headaches, neurocognitive difficulties, depression, muscle pains, gastrointestinal disorders, rashes, metabolic disruption, and thromboembolic conditions. Long-term COVID-19 symptoms are most associated with skeletal muscle, gut, and brain issues. Research has shown that several comorbidities affect a patient's response to COVID-19 infection, such as nutritional status, chronic diseases, and age.

Research conducted by the NIH has suggested that transcobalamin plays a role in the suppression of systemic inflammation, specifically by reducing the levels of interleukin-6 and other inflammatory growth factors [4]. B12 also functions to regulate nitric oxide, which is a regulator of the immune response to infection, and in turn, decreases the concentration of nuclear transcription factor kB (NFkB). At the same time, B12 works to boost the immune system by increasing the number of CD8+ T cells and natural killer T cells present in the body [4].

Low B12 levels are also correlated with high homocysteine levels due to the lack of breakdown of homocysteine into methionine. Homocysteine has been seen to increase the production of reactive oxygen species during lipid peroxidation and endothelial damage. This, in turn, increases the potential for an embolic event to occur [4].

There is research evaluating the relationship of the skeletal muscle-gut-brain axis, with the idea that these three systems have an intimate relationship with one another. Insulin-resistant skeletal muscle can lead to fatigue and decreased exercise. This can lead to an imbalance in the microbiome of the gut, which can decrease vagal tone [4]. Vagal tone is important in maintaining brain health; therefore, a decrease can contribute to the development of psychiatric and neurodegenerative diseases [4]. Additionally, if there is an imbalance in the gut microbiome, it can increase the number of lipopolysaccharides and interleukins, leading to skeletal muscle insulin resistance and psychiatric and neurodegenerative diseases [4]. These are symptoms seen with COVID-19 that could be ameliorated with vitamin B12 supplementation.

Vitamin B12 supplementation in conjunction with COVID-19 treatment is still controversial, and ongoing research is being conducted. While many studies conclude that B12 can reverse several of the symptoms, a few studies have shown no benefit, and others have shown an inverse relationship. However, according to the NIH, these studies also listed limitations that could have impacted their results [4]. Therefore, it is recommended to check patients' B12 levels who have infections to confirm that they are within a proper clinical range [4]. 

Recommendations and limitations

Based on the information reviewed, methylcobalamin appears to be the more effective and bioavailable form of vitamin B12, especially for individuals with conditions that impair absorption or methylation. It may be beneficial to screen for B12 deficiency in at-risk populations, such as the elderly, those with GI disorders, or individuals following strict vegetarian or vegan diets. Earlier detection and treatment could help prevent long-term neurologic and hematologic complications.

That said, this paper is a literature review and not a clinical trial, so it relies heavily on previously published data. This makes it harder to control for bias across studies or account for population-specific differences. There’s still a need for more large-scale, comparative studies such as meta-analyses that explore different forms of B12 supplementation in various groups, especially those with chronic illness or genetic variants affecting metabolism.

## Conclusions

In conclusion, vitamin B12 plays a vital role in human health. It serves essential roles in DNA synthesis, red blood cell formation, myelination, and enzymatic functions. Though naturally available in animal products, a variety of populations, including vegans, the elderly, and those with absorption disorders, may need supplementation. Evidence shows that both synthetic forms, like cyanocobalamin, and natural forms, such as methylcobalamin, can effectively raise serum B12 levels, and supplementation has shown promise in alleviating symptoms of neurologic and psychiatric disorders related to B12 deficiency. Therefore, understanding the bioavailability, mechanisms of absorption, and therapeutic roles of varying forms of B12 is essential in both preventative and clinical care. Considering this, this paper aims to gather information on susceptible groups and determine whether natural B12 or B12 in supplement form is more beneficial, either through ease of access or absorption. At the same time, key questions remain: Is there further research to conduct to make B12 digestion and absorption faster? Are there long-term benefits or consequences to B12 supplementation? Should B12 supplementation be given prophylactically to susceptible groups? Further investigation is needed to address these questions.
